# Supplementary material for: Improving measurement of functional status among older adults in primary care: A pilot study
Source: PLoS One. 2024 May 13;19(5):e0303402. doi: 10.1371/journal.pone.0303402 (PMC11090365; doi:10.1371/journal.pone.0303402)
Supplement: Attachment — Submitted filename: Comments PLOS One - DJS.pdf [file pone.0303402.s001.pdf]

Comments PLOS One

Dear Editor,

This article deals with a very interesting topic. The relevance of this article is undeniable. Additionally, the article is well structured, the methods of analysis are relevant, and the tables are informative. However, before its publication, some changes need to be made.

You will find my comments below.

Sincerely yours

In the fidelity section on page 32, you should be careful with confidence intervals given your sample (n=21). I therefore suggest that the authors give the limits of these results.
