# Supplementary material for: Improving measurement of functional status among older adults in primary care: A pilot study
Source: PLoS One. 2024 May 13;19(5):e0303402. doi: 10.1371/journal.pone.0303402 (PMC11090365; doi:10.1371/journal.pone.0303402)
Supplement: Attachment — Submitted filename: Response to Reviewers.docx [file pone.0303402.s002.docx]

Rebecca T. Brown

Ralston House

3615 Chestnut Street

Philadelphia, PA 19104

telephone: (215) 573-2021

e-mail: Rebecca.Brown@pennmedicine.upenn.edu

February 12, 2024

De-Chih Lee, Ph.D.
Academic Editor
PLOS ONE

Dear Dr. Lee,

We thank the Editor and Reviewers for their helpful comments and appreciate the opportunity to address the comments and revise our manuscript. Below, please find item-by-item responses to the comments, which are included verbatim. All page and paragraph numbers refer to locations in the revised manuscript. We have uploaded a clean copy of the revised manuscript as well as a copy of the manuscript with tracked changes.

**Responses to Journal Requirements:**

1. **Comment:** Please ensure that your manuscript meets PLOS ONE's style requirements, including those for file naming. The PLOS ONE style templates can be found at
   [https://journals.plos.org/plosone/s/file?id=wjVg/PLOSOne_formatting_sample_main_body.pdf](https://urldefense.com/v3/__https:/journals.plos.org/plosone/s/file?id=wjVg*PLOSOne_formatting_sample_main_body.pdf__;Lw!!LQC6Cpwp!sokIgrCR_8T4tKUnmxQl2dOVkNpRibCRstICTrNoFEMUE-7h31aUZIhUb5-vr-11lvmvhURrW2ryL-CYftqrSkmIjfT2$) and 
   [https://journals.plos.org/plosone/s/file?id=ba62/PLOSOne_formatting_sample_title_authors_affiliations.pdf](https://urldefense.com/v3/__https:/journals.plos.org/plosone/s/file?id=ba62*PLOSOne_formatting_sample_title_authors_affiliations.pdf__;Lw!!LQC6Cpwp!sokIgrCR_8T4tKUnmxQl2dOVkNpRibCRstICTrNoFEMUE-7h31aUZIhUb5-vr-11lvmvhURrW2ryL-CYftqrSk1rV_YW$)
   a. **Response:** We have formatted the manuscript to meet PLOS ONE’s style and naming requirements.
2. **Comment:** Note from Emily Chenette, Editor in Chief of PLOS ONE, and Iain Hrynaszkiewicz, Director of Open Research Solutions at PLOS: Did you know that depositing data in a repository is associated with up to a 25% citation advantage ([https://doi.org/10.1371/journal.pone.0230416](https://urldefense.com/v3/__https:/doi.org/10.1371/journal.pone.0230416__;!!LQC6Cpwp!sokIgrCR_8T4tKUnmxQl2dOVkNpRibCRstICTrNoFEMUE-7h31aUZIhUb5-vr-11lvmvhURrW2ryL-CYftqrStUAvY_X$))? If you’ve not already done so, consider depositing your raw data in a repository to ensure your work is read, appreciated and cited by the largest possible audience. You’ll also earn an Accessible Data icon on your published paper if you deposit your data in any participating repository ([https://plos.org/open-science/open-data/#accessible-data](https://urldefense.com/v3/__https:/plos.org/open-science/open-data/*accessible-data__;Iw!!LQC6Cpwp!sokIgrCR_8T4tKUnmxQl2dOVkNpRibCRstICTrNoFEMUE-7h31aUZIhUb5-vr-11lvmvhURrW2ryL-CYftqrSvXVcDLk$)).
   a. **Response:** We now clarify that this study was funded in 2015, before the U.S. government developed guidance for studies funded by the VA to publicly share underlying data. For this reason, consent documents for participants in this study did not include ethical approval to publicly share underlying data, and public deposition of these data would breach compliance with the protocol approved by our research ethics boards.
3. **Comment:** Please describe in your methods section how capacity to provide consent was determined for the participants in this study. Please also state whether your ethics committee or IRB approved this consent procedure. If you did not assess capacity to consent please briefly outline why this was not necessary in this case.
   a. **Response:** Among patients, we assessed ability to provide informed consent using a teach-back method to ensure understanding of the key elements of informed consent. We did not assess ability to provide informed consent among staff members, since they are not considered a population at risk for lacking capacity to provide informed consent. This consent procedure was approved by our IRB.
   b. **Methods, page 9, paragraph 1:** “All participants provided verbal consent to participate in the evaluation, which was witnessed and documented by research staff in an electronic database. Among patients, we assessed ability to provide informed consent using a teach-back method.(41) We did not assess ability to provide informed consent among staff members, since they are not considered a population at risk for lacking ability to provide informed consent. These consent procedures were approved by our IRB.”
4. **Comment:** In the ethics statement in the Methods, you have specified that verbal consent was obtained. Please provide additional details regarding how this consent was documented and witnessed, and state whether this was approved by the IRB.
   a. **Response:** Verbal consent was witnessed and documented by research staff in the study’s electronic database. This approach was approved by the IRB. Please see response #3 above for the added text to the Methods.
5. **Comment:** In the online submission form, you indicated that "The underlying data for this study consists of surveys and in-depth, qualitative interviews with (1) older Veterans and (2) employees of the U.S. Veterans Health Administration. It is not possible to create a minimal data set with this survey and qualitative data as this study did not obtain ethical approval or informed consent from participants to publicly share underlying survey and qualitative data sets. Relevant excerpts from transcripts are included within the paper. The datasets generated and/or analyzed during this study are not publicly available but may be available upon request at the Center for Health Equity Research and Promotion of the U.S. Department of Veterans Affairs administrative offices, at 215-823-5817.” All PLOS journals now require all data underlying the findings described in their manuscript to be freely available to other researchers, either 1. In a public repository, 2. Within the manuscript itself, or 3. Uploaded as supplementary information. This policy applies to all data except where public deposition would breach compliance with the protocol approved by your research ethics board. If your data cannot be made publicly available for ethical or legal reasons (e.g., public availability would compromise patient privacy), please explain your reasons on resubmission and your exemption request will be escalated for approval.
   a. **Response:** We now clarify that this study was funded in 2015, before the U.S. government developed guidance for studies funded by the VA to publicly share underlying data. For this reason, consent documents for participants in this study did not include ethical approval to publicly share underlying data, and public deposition of these data would breach compliance with the protocol approved by our research ethics boards. We have updated the language in the statement as follows to include a description of both the qualitative and quantitative data.
   b. “The underlying data for this study consists of (1) in-depth, qualitative interviews with (a) Veterans with aging-related functional impairment and their caregivers and (b) employees of the U.S. Veterans Health Administration; and (2) quantitative measures of functional status among Veterans. For both the qualitative and quantitative data, it is not possible to create a minimal data set as this study did not obtain ethical approval or informed consent from participants to publicly share underlying data sets. For the qualitative data, relevant excerpts from transcripts are included within the paper. The qualitative and quantitative datasets generated and/or analyzed during this study are not publicly available but may be available upon request at the Center for Health Equity Research and Promotion of the U.S. Department of Veterans Affairs administrative offices, at [215-823-5817](mailto:215-823-5817).”
6. **Comment:** Please review your reference list to ensure that it is complete and correct. If you have cited papers that have been retracted, please include the rationale for doing so in the manuscript text, or remove these references and replace them with relevant current references. Any changes to the reference list should be mentioned in the rebuttal letter that accompanies your revised manuscript. If you need to cite a retracted article, indicate the article’s retracted status in the References list and also include a citation and full reference for the retraction notice.
   a. **Response:** We have reviewed our reference list to ensure that it is complete and correct, and have added several references suggested by the Reviewers, as discussed below. We have ensured that the list does not include retracted papers.

**Responses to Editor:**

1. **Comment:** Please make minor revisions according to the comments of the two reviewers.
   a. **Response:** We thank the Editor and have responded to each Reviewer comment below.

**Response to Reviewer #1:**

1. **Comment:** Dear Editor,
   This article deals with a very interesting topic. The relevance of this article is undeniable. Additionally, the article is well structured, the methods of analysis are relevant, and the tables are informative. However, before its publication, some changes need to be made.
   You will find my comments below.
   Sincerely yours
a. **Response:** We thank the Reviewer for this comment and have responded in detail below.
2. **Comment:** In the fidelity section on page 32, you should be careful with confidence intervals given your sample (n=21). I therefore suggest that the authors give the limits of these results.
   a. **Response:** We appreciate the Reviewer’s comment and have added text to acknowledge these limitations in the Discussion, as follows:
   b. **Discussion, page 35, paragraph 3:** “The sensitivity and specificity of the two-step screening approach for detecting ADL and IADL impairment compared to full screening ranged from fair to very good, although confidence intervals were wide given the small sample size.”

**Response to Reviewer #2:**

1. **Comment:** Thank you for the opportunity to review this excellent manuscript on improving measurement of functional status among older adults in primary care. This is a well written paper on an important topic that will be of great interest to readers seeking way to improve care of older adults. Please consider the following suggestions which may strengthen this manuscript:
   a. **Response:** We thank the Reviewer for these comments and have responded to specific comments below.
2. **Comment:** - Introduction - consider using the term 'person centered' instead of 'patient centered' (see here [https://www.ncbi.nlm.nih.gov/pmc/articles/PMC6069658/](https://urldefense.com/v3/__https:/www.ncbi.nlm.nih.gov/pmc/articles/PMC6069658/__;!!LQC6Cpwp!sokIgrCR_8T4tKUnmxQl2dOVkNpRibCRstICTrNoFEMUE-7h31aUZIhUb5-vr-11lvmvhURrW2ryL-CYftqrSlDAZRtq$))
   a. **Response:** We appreciate the Reviewer’s suggestion and have edited the manuscript accordingly.
3. **Comment:** - consider adding understanding frailty after 'assessing prognosis'
   a. **Response:** We appreciate the Reviewer’s suggestion and have added this text to the Introduction (page 5, paragraph 1).
4. **Comment:** - page 5, paragraph two - consider giving an example of routine outpatient screenings in the VA (do you mean things like PHQ2 for depression?
   a. **Response:** We have added examples of routine screenings implemented and evaluated in the VA, as follows:
   b. **Introduction, page 5, paragraph 2:** “The Veterans Health Administration (VA), the largest integrated health system in the United States (US), is a leader in implementing and evaluating a range of routine screening and assessment efforts in outpatient settings, such as screening for alcohol misuse and housing instability [30-32].”
5. **Comment:** - page 9 - para 2 - was it any kind of clinica appointment or only routine PCP (vs urgent visit)?
   a. **Response:** Because of the way screeners are triggered within the VA electronic health record, the screener is available for any type of visit with the PCP, either urgent or routine. We have clarified this point in the Methods as follows:
   b. **Methods, page 9, paragraph 2:** “Patients who had a clinic appointment scheduled with their PCP (routine or urgent visit) during the trial period and had the electronic questionnaire administered by clinic staff were eligible to participate in an interview about their experience with the intervention.”
6. **Comment:** - page 13 - would specific on the last line that it was pandemic related restrictions that limited ethnographic observations
   a. **Response:** We appreciate the Reviewer’s suggestion and have added this information, as follows:
   b. **Results, page 13, paragraph 2:** “We planned to assess fidelity via ethnographic observation, but this was not possible due to pandemic-related restrictions on in-person visits.”
7. **Comment:** Methods - this is so clearly delineated and easy for the reader to follow, thank you!
   a. **Response:** We thank the Reviewer for this comment.
8. Would describe a bit about what the educational session entailed (maybe as a supplemental table that outlines the session?)
   a. **Response:** We have now updated Figure 1 to include more details on the components of the educational session (see updated figure). Of note, based on feedback from participants in our formative evaluation, the session was quite brief (~10 minutes) and focused on (1) providing evidence for the importance of functional status measurement, (2) reviewing the core components of the intervention, and (3) sharing key findings from our formative interviews with patients and caregivers (e.g., the importance of measuring both difficulty and need for help with daily activities).
9. **Comment:** Results
   Table 2 - in the legend would list what you included in your definition of ADLs (Bathing etc) and iADLS
   a. **Response:** We appreciate the Reviewer’s comment and have edited Table 2 as suggested.
10. **Comment:** page 22 - can you give an example of the types of practice changes participants reported intending to try?
    a. **Response:** We have clarified that practice changes included making more time for functional status assessment and using the results of the screener to make patient referrals, as follows:
    b. **Results, page 21, paragraph 2:** “In open-ended responses, LVNs and PCPs reported several plans to change their professional practice in response to the educational session. Planned changes included feeling “more motivated” to make time for functional status assessment and using the results of the screener to “refer patients to specific services,” including geriatrics and rehabilitation.”
11. **Comment:** The results section is easy to follow and clearly reported.
    a. **Response:** We thank the Reviewer for this comment.
12. **Comment:** Discussion:
    - consider explaining why you picked age 75 and older for the intervention and how that might affect prevalence of functional impairment in your pilot
    a. **Response:** We now clarify that we used this age cut-off because this is the age cut-off currently used by the VA Office of Geriatrics & Extended Care (GEC) for screening for geriatric syndromes (and because both GEC and primary care requested that we use this cut-off to limit screening burden); because the prevalence of functional impairment increases with age, this may have resulted in a higher prevalence of functional impairment in this pilot than in a study using a younger age cut-off. We have edited the Discussion as follows:
    b. **Discussion, page 37, paragraph 2:** “We used an age cut-off of 75 and older for the intervention in order to align with existing VA screening measures among older adults. Because the prevalence of functional impairment increases with age, this may have resulted in a higher prevalence of functional impairment than in a study using a younger age cut-off.”
13. **Comment:** - page 35 last line - again consider 'person-centered' instead of patient centered
    a. **Response:** We have edited the manuscript as suggested.
14. **Comment:** - page 36, first paragraph - what do you mean by "just need for help"?
    a. **Response:** We have rephrased this sentence as follows to clarify that the screener identifies individuals who have difficulty performing ADLs and IADLs but do not yet need help with these tasks:
    b. **Discussion, page 35, paragraph 1:** “This study also provides an approach to identify earlier stages of functional impairment (i.e., when participants have difficulty performing daily activities but do not yet need help) [58]. Earlier identification could allow for earlier intervention, with the potential to help prevent or delay subsequent functional decline and associated health care utilization.”
15. **Comment:** - page 36 - second last para, the adoption and reach repeats nearly verbatim what's in the opening of the discussion so would rephrase
    a. **Response:** We appreciate this suggestion and have edited the manuscript as follows:
    b. **Discussion, page 35, paragraph 2:** “Consistent with the high acceptability of the intervention, adoption by LVNs and PCPs was high, with all participating LVNs completing screening for functional impairment and nearly half of patients with identified impairments receiving referrals within 1 week of screening.”
16. **Comment:** - consider mentioning here or in the intro the importance of this work to the VA's goal of becoming an Age-Friendly health system (could cite [https://agsjournals.onlinelibrary.wiley.com/doi/10.1111/jgs.18070](https://urldefense.com/v3/__https:/agsjournals.onlinelibrary.wiley.com/doi/10.1111/jgs.18070__;!!LQC6Cpwp!sokIgrCR_8T4tKUnmxQl2dOVkNpRibCRstICTrNoFEMUE-7h31aUZIhUb5-vr-11lvmvhURrW2ryL-CYftqrSkN8fS4B$)and/or [https://www.ncbi.nlm.nih.gov/pmc/articles/PMC10204932/](https://urldefense.com/v3/__https:/www.ncbi.nlm.nih.gov/pmc/articles/PMC10204932/__;!!LQC6Cpwp!sokIgrCR_8T4tKUnmxQl2dOVkNpRibCRstICTrNoFEMUE-7h31aUZIhUb5-vr-11lvmvhURrW2ryL-CYftqrSvmJuj1x$)
    a. **Response:** We have edited the manuscript to include this point and these references, as follows:
    b. **Discussion, page 38, paragraph 1:** “Proactively measuring functional status has the potential to improve identification and management of functional status for older adults on a broad scale, leading to improvements in population health, consistent with VA’s goal of becoming the largest Age-Friendly Health System in the United States [68,69].”
17. **Comment:** This is such a well written article on such an important topic - thank you for the incredible work you are doing!
    a. **Response:** We appreciate the Reviewer’s comment.

We thank the Editor and Reviewers for their time and efforts to improve our manuscript.

Sincerely,

Rebecca Brown
