# Supplementary material for: Improving measurement of functional status among older adults in primary care: A pilot study
Source: PLoS One. 2024 May 13;19(5):e0303402. doi: 10.1371/journal.pone.0303402 (PMC11090365; doi:10.1371/journal.pone.0303402)
Supplement: Attachment — Submitted filename: PLOS One Response letter 3.27.24.docx [file pone.0303402.s003.docx]

Rebecca T. Brown

Ralston House

3615 Chestnut Street

Philadelphia, PA 19104

telephone: (215) 573-2021

e-mail: Rebecca.Brown@pennmedicine.upenn.edu

March 27, 2024

De-Chih Lee, Ph.D.
Academic Editor
PLOS ONE

Dear Dr. Lee,

Below, please find item-by-item responses to the journal requirements, which are included verbatim.

**Responses to Journal Requirements:**

1. **Comment:**

In the online submission form, you indicated that 

"The underlying data for this study consists of (1) in-depth, qualitative interviews with (a) Veterans with aging-related functional impairment and their caregivers and (b) employees of the U.S. Veterans Health Administration; and (2) quantitative measures of functional status among Veterans. For both the qualitative and quantitative data, it is not possible to create a minimal data set as this study did not obtain ethical approval or informed consent from participants to publicly share underlying data sets. For the qualitative data, relevant excerpts from transcripts are included within the paper. The qualitative and quantitative datasets generated and/or analyzed during this study are not publicly available but may be available upon request at the Center for Health Equity Research and Promotion of the U.S. Department of Veterans Affairs administrative offices, at 215-823-5817."

All PLOS journals now require all data underlying the findings described in their manuscript to be freely available to other researchers, either 
1. In a public repository, 
2. Within the manuscript itself, or 
3. Uploaded as supplementary information.

This policy applies to all data except where public deposition would breach compliance with the protocol approved by your research ethics board. If your data cannot be made publicly available for ethical or legal reasons (e.g., public availability would compromise patient privacy), please explain your reasons on resubmission and your exemption request will be escalated for approval."

a. **Response:** We have clarified that this study was funded in 2015, before the U.S. government developed guidance for studies funded by the VA to publicly share underlying data. For this reason, consent documents for participants in this study did not include ethical approval to publicly share underlying data, and public deposition of these data would breach compliance with the protocol approved by our research ethics boards. We have previously provided language describing this, as quoted below (italics added for emphasis). We are requesting that our exemption request be escalated for approval.

"The underlying data for this study consists of (1) in-depth, qualitative interviews with (a) Veterans with aging-related functional impairment and their caregivers and (b) employees of the U.S. Veterans Health Administration; and (2) quantitative measures of functional status among Veterans. *For both the qualitative and quantitative data, it is not possible to create a minimal data set as this study did not obtain ethical approval or informed consent from participants to publicly share underlying data sets.* For the qualitative data, relevant excerpts from transcripts are included within the paper. The qualitative and quantitative datasets generated and/or analyzed during this study are not publicly available but may be available upon request at the Center for Health Equity Research and Promotion of the U.S. Department of Veterans Affairs administrative offices, at 215-823-5817."

We thank the Editor for their time and efforts to improve our manuscript.

Sincerely,

Rebecca Brown
